# Assessment of Risk of Bias in Randomized Trials Using RoB 2: Reflections From a Systematic Review of Complex Interventions

**DOI:** 10.1002/cesm.70107

**Published:** 2026-10-21

**Authors:** Preethy D'Souza, Wendy Macdowall, Helen E. D. Burchett, Irene Kwan, Katy Sutcliffe

**Affiliations:** ^1^ EPPI Center University College London London UK; ^2^ Faculty of Public Health & Policy London School of Hygiene and Tropical Medicine London UK

**Keywords:** Cochrane RoB 2, evidence appraisal, quality appraisal, risk of bias, systematic reviews

## Abstract

The Cochrane risk‐of‐bias tool (RoB 2) is used to assess the risk of bias in randomized trials in systematic reviews. However, studies have shown that the tool is often incorrectly used and published reviews rarely include detail regarding how it was applied or offer reflections on using it. This paper is a transparent applied case example with lessons learned and limitations of using RoB 2 in a (non‐Cochrane) systematic review evaluating the effectiveness of remote therapies for alcohol and drug use disorders. We share our reflections on aspects to plan in advance (pre‐selecting the outcome(s) and their results and piloting the tool); factors to consider when applying the tool (multiple outcomes within a study and navigating resources); overcoming common problems within specific domains (developing a review‐specific guidance document). Through our worked example, this article illustrates how review teams can engage with the RoB 2 tool within a specific review context to enhance its correct application.

## 1 Introduction

The Cochrane Risk of Bias (RoB) tool is commonly used for appraising randomised controlled trials (RCTs) in both Cochrane and non‐Cochrane reviews [1]. The revised version (RoB 2) [2] aims to increase the reliability of assessments. Assessments are made for a particular outcome rather than the whole study. RoB 2 assesses risk of bias across five domains: (1) the randomization process, (2) deviations from the intended intervention, (3) missing outcome data, (4) outcome measurement, and 5) reporting of results. There are several ‘signaling’ questions within each domain, and algorithms to support assessment of whether a study's outcome is at low risk of bias, points to some concerns, or is at high risk of bias for each domain, as well as overall. It encompasses a broader range of issues than its predecessor including post‐randomization deviations from the intended interventions. It also incorporates advances in estimation of intervention effects (e.g., per‐protocol and intention‐to‐treat analyzes) and theoretical discussion of bias (e.g., types of bias, biases arising at different stages of a trial) [3, 4].

Since RoB 2 became available in 2019, several studies have highlighted challenges and issues in using it [5, 6, 7, 8, 9, 10]. One study examined adherence to RoB 2 guidance in 208 published reviews. The authors found that adherence could not be assessed in 34% of reviews, either because they used the original RoB tool despite stating in the methods that they used RoB 2 – or the review authors did not report the number of primary outcomes preventing the authors of this study from evaluating the adherence to RoB2 guidance. Among reviews that could be assessed, adherence was 69%, however, in reviews with more than one primary outcome adherence dropped to 29% [5]. A 2024 study [6] of 267 non‐Cochrane reviews found that fewer than one in ten presented a complete RoB 2 assessment and in most reviews (81.7%) risk of bias was incorrectly assessed at the study level rather than the outcome level.

Minozzi et al. (2020) [7] assessed interrater reliability (IRR) among four independent assessors applying RoB 2 to outcomes from 70 RCTs. Findings indicated poor agreement between assessors (IRR 0.16, 95% confidence interval (CI) 0.08–0.24). Specific challenges included different approaches taken between assessors for some domains and difficulties in understanding new terminology and the way signaling questions were formulated. A follow‐up article by the same author revealed that the development and use of a review‐specific implementation document improved reliability (IRR 0.11 – 0.42) [10]. While these evaluations highlight issues with its application, the published reviews on which they are based rarely include details of how RoB 2 was applied or reflections on using it. We found a recent publication about a tool (ROBUST‐RCT) and the authors reported that the tool is developed to overcome some of the challenges of existing tools and is rigorously developed, simply structured, and user‐friendly [11]. Cochrane editors published ‘top tips’ [12] for successfully applying RoB 2 based on their observations of common errors in its use in Cochrane reviews. They concluded that advice from the user perspective would also be valuable for supporting reviewers to use the tool with confidence and assess the risk of bias accurately. In this article, we share our reflections as users of RoB 2 in a non‐Cochrane systematic review of complex interventions, including lessons learned and limitations. Our review addressed the question ‘How effective are interactive remote therapies for reducing alcohol/drug use?’ [13].

### 1.1 Using RoB 2 in Our Review

We had a total of 34 RCTs in our meta‐analysis which reported 42 interventions and 7 RCTs measured both the outcomes. In total, 49 results from 42 interventions were assessed for RoB. The two selected outcomes were relapse (i.e., abstinence not maintained), for which the result was the odds of relapse, and for days of use, the result was the standardized mean difference in days of use, measured by self‐report and/or toxicology [14]. Our RoB 2 assessments targeted the effect of assignment to intervention as we found it appropriate for the nature of interventions and the intended purpose of the review. All review team members were experienced systematic reviewers, with prior experience in critical appraisal. However, all but one (PD) were using RoB 2 for the first time. We used EPPI‐Reviewer software to carry out the assessments [15].

We applied RoB 2 to the full trial reports, linked articles and published protocols (where available) using the cribsheet [16], the full guidance document [17] and instructional videos [18].

In the following sections, we illustrate our experience and the strategies we employed to optimize the application of the tool. We use the three main areas identified by Cochrane editors to structure our reflections [12], namely aspects to plan in advance, factors to consider when applying the tool and overcoming common problems within specific domains.

## 2 Aspects to Plan in Advance

### 2.1 Strategies for Preselecting Results for Synthesis

Since assessments are result‐specific rather than conducted at the study level, reviewers must decide which results to synthesize before they can commence with applying RoB 2. To avoid potential bias, Cochrane guidance directs review teams to prespecify which results will be synthesized by indicating the outcome domains and ideally outcome measures in their protocol [2]. However, in practice many review teams do not specify at the protocol stage precisely which results will be synthesized [12]. When consensus exists about core outcomes in a field, specifying at the protocol stage may be possible. In fields where no such consensus exists ‐ such as for alcohol/drug use disorder treatment interventions [19, 20] specification at the protocol stage can be problematic. Doing so risks a review with few included studies, or an empty review, or may preclude the possibility of conducting a meta‐analysis when one could have taken place had other relevant outcome measures been selected. Whilst assessing all measured outcomes and their results would overcome these risks, Cochrane recognizes that “*it is unlikely to be feasible to assess every result for every trial in the review*” (section 8.2.1) [2]. RoB 2 guidance notes that “*We have not yet formulated recommendations on which results should be targeted with an assessment, or how many results should be assessed*” (page 6) [17]. However, authors are encouraged to select which specific results from the included trials to synthesize (and therefore to assess for RoB) and focuses on the main outcomes of the review; the results contributing to the review's ‘Summary of findings’ table in the Cochrane reviews.

We developed our protocol [21] in consultation with stakeholders and, following their advice, we pre‐specified the outcome domains, rather than specific outcomes and results, that would be included in our review. We specified that we would include any quantitative substance use measures, as opposed to measures in other domains such as mental health or criminal justice involvement. Therefore, we sought a transparent method to select results for synthesis that was unbiased (i.e., independent of the magnitude or direction of effect estimates) but that would maximize our chances of conducting a statistical synthesis. We developed the following three‐step selection method to identify specific results for synthesis and RoB assessment.

### Step 1 – Selecting the Most Frequently Measured Outcomes

To maximize the number of clinically relevant comparable results, we decided to identify and select the most frequently measured alcohol/drug use outcomes across the studies; these were relapse (i.e., abstinence not maintained) and days of use. To minimize the risk of selective reporting bias, arising from studies being less likely to report non‐statistically significant results, eligibility was based on whether the authors reported measuring the relevant outcome, rather than whether they actually reported the result or provided complete information about it [22]. (Note: Although our selection approach is not inherently prone to selective reporting bias, we assessed whether our review was affected by unreported results or missing details. Some studies lacked sufficient information to calculate an effect size, but this was due to missing standard deviations or results reported only as part of a meta‐regression, rather than selective reporting based on their P value, magnitude, or direction. We reported these results narratively. We did not, however, formally apply the ROB‐ME tool [22] to assess selective reporting bias, which is a limitation of our approach.)

This selection method was also applied alongside other criteria such as robustness (see step 2), relevance to stakeholders and value to policy and practice, since frequency of measurement should not be a standalone approach.

### Step 2 – Prioritizing the Most Robust Measures

Both relapse and days of use were measured by different means in the included trials. Some used self‐report (validated or non‐validated tools), others used toxicology (urine or breath analysis), and some used both. When a study used more than one means to measure a particular outcome, we selected the most robust measure available, that is, we selected toxicology, or self‐report verified by toxicology, over self‐reported outcomes. If only self‐reported data was available, we selected those measured using validated tools for example TLFB (Timeline Follow Back) [23] over non‐validated tools.

### Step 3 – Selecting Comparable Time Points

The duration of the intervention(s) in the included trials varied considerably, and outcomes were measured at different time points. The longest timepoint would have provided the most clinically relevant and meaningful outcome data about the long‐term effects of interventions, but relatively few reported follow‐up data at comparable timepoints. We therefore decided that for comparability we would extract end‐of‐intervention outcomes, or the nearest time point to that.

### Suggestions for Supporting Unbiased Outcome Selection When It Has Not Been Pre‐Specified

Since many reviewers will face the challenge of specifying outcomes, measures and timepoints for synthesis as they commence RoB 2 assessments, it would be helpful if RoB 2 documents could direct reviewers to guidance on unbiased approaches for this. At the protocol stage, if it is challenging to select outcomes and the results, review teams could instead predefine a selection method that protects against bias but enables reviewers to maximize data for synthesis (e.g., pre‐specifying that the outcome with robust measures will be selected).

### 2.2 Piloting the Tool and Ongoing Discussion

As recommended by the Cochrane editors [12], we piloted the tool in our review. To ensure we applied the tool consistently, all team members first independently assessed a sample of outcomes and compared assessments to arrive at consensus. The remaining assessments were conducted in pairs. We continued to meet regularly as a team to discuss and develop consensus about emerging issues to ensure consistency within and across the 49 assessments as we worked through them, keeping a record of the decisions made. There were instances when we had to revisit our assessments after team discussions. We also developed a review‐specific guidance document, as suggested by the Cochrane editors, which we found to be critically important for supporting consistency [12].

### Suggestions on Piloting and Ongoing Discussion

We agree that piloting RoB 2 on a sample of outcomes is vital. However, we found ongoing discussion within the review team was also essential to address emerging issues, develop the review‐specific guidance document as needed, and, if necessary, revisit prior assessments.

## 3 Factors to Consider When Applying the Tool

### 3.1 Using Tool on Multiple Outcomes Within a Single Study

Our review included seven trials that needed to be assessed twice, as they reported both of our selected outcomes, and RoB 2 assessments are result‐specific. For example, in our review, we recognized that retrospective self‐reported alcohol/drug use data is more susceptible to biases ‐ due to recall and social desirability ‐ than toxicology data. However, during the assessments, we found that domain 1 (bias arising from the randomization process) could be applied across multiple results within the same study. We also found that some of the information captured for signaling questions in domain 2 (deviations from the intended intervention) could be reused across results, however the final assessment remains result‐specific. Domains 3 (missing outcome data), 4 (outcome measurement) and 5 (selection of reported result) are result ‐specific and so each relevant result will need to be assessed.

### Suggestion for Multi‐Outcome Assessments Within a Single Study

To avoid duplication of effort, it would be useful if RoB 2 guidance mentioned that the review team could identify which information could be reused across outcomes that need not be repeated in assessments of multiple outcomes from a single study and which do.

### 3.2 Navigating the Resources

Multiple RoB 2 resources are available to reviewers, including a full guidance document [17], training videos and webinars [18], and a crib sheet [16]. The Cochrane editors acknowledge that assessing risk of bias is inherently complex and that users need to be familiar with the RoB 2 guidance documents [12]. The application of RoB 2 to our set of trials necessitated frequent referral to all these resources. Whilst the crib sheet provided a helpful overview, we often found it lacked sufficient detail to make an assessment. The training videos offered additional insights not available in either the full guidance or the crib sheet. However, we found that it was not always clear which resource would provide the required additional information, which made navigating these resources particularly time‐consuming. A recent publication by Tomlinson et al [24] also reported that the guidance is complex and its application is demanding, and it lacks the specificity sufficient to operationalize it. While the full guidance, crib sheet and template were compiled on one webpage [25], the starter pack with links to relevant sections of the Cochrane handbook, FAQs, and webinars was hosted on another [26].

### Suggestions for Navigating the RoB 2 Guidance Documents

Reflecting on our experience and in line with the recommendations of the Cochrane editors [12], we suggest that review teams familiarize themselves with the various RoB 2 guidance documents to facilitate their use during risk of bias assessments. Consolidating guidance into a single, multi‐layered interactive resource would make it easier to delve deeper into explanations and examples when needed. For example, within the crib sheet, links could be inserted that take reviewers directly to the relevant sections of the longer guidance document, the specific video, or section of a video and to examples or other relevant resources.

## 4 Overcoming Common Problems Within Specific Domains

### 4.1 Developing a Review Specific Guidance Document

Despite referring to multiple sources for each outcome assessment (including the protocol and linked articles where available), explicit information to enable assessments was often unavailable or unclear, and judgment was needed in the assessment. In our review, selective reporting was the domain for which information was most frequently unavailable. Our approach was to answer ‘No information’ when analysis intentions were not available in a protocol or pre‐registered trial registration.

Cochrane editors, in their Tips 6–10, emphasized that review teams should make reasonable assumptions and provide justifications based on the trial context in areas such as blinding, deviation from intended interventions, and missing outcome data, to ensure that trials receive the risk‐of‐bias judgments they deserve [12]. For transparency and to ensure consistency in assessment, we recorded all our interpretations of the questions in the context of our review in a ‘bespoke guidance’ document. Examples of how we developed review‐specific guidance, and its implications for assessment, are illustrated in Table 1. This aligns with the Cochrane editors’ recommendation to develop a guidance document outlining group‐made decisions that can be used by all team members when assessing risk of bias. Specific guidance tailored to the review to help to overcome challenges and ensure consistency across assessments was also highlighted by Tomlinson et al. [24].

**Table 1:** Examples of selected signaling questions and how we applied RoB 2 in our review context.

Signaling question	Our judgment and implications for RoB 2 assessment
**2.2. Were carers and people delivering the interventions aware of participants' assigned intervention during the trial?**	**Judgment:** In the absence of an explicit statement of the trial being ‘open’ or ‘closed’, where people delivering the intervention had an active role ‐ such as training participants to use digital/computer programs ‐ we felt it was reasonable to assume that providers were probably aware of assignment, therefore we coded these as ‘Yes/Probably yes’. **Implication:** Answering ‘Yes/Probably yes’ meant that the outcome could only be assessed as ‘Some concerns’ or ‘High risk’ for domain 2.
**2.3. Were there deviations from the intended intervention that arose because of the trial context?**	**Judgement:** We felt that it was a reasonable assumption that, in our included trials, it was unlikely that participants in the control group would switch arms to access the intervention. There is the possibility of other types of deviations, such as trial personnel implementing non‐protocol interventions, failure to implement the intended interventions, and non‐adherence by trial participants to their assigned intervention, but we did not find any evidence of this in the reports of our included trials. Therefore, unless we identified specific concerns, we coded this as, ‘No/Probably no’. **Implication:** Answering ‘No/Probably no’ rather than ‘No information’ meant outcomes could be assessed as ‘Low risk’ rather than ‘Some concerns’ which we felt was more appropriate in our context.
**3.2 If N/PN/NI to 3.1: Is there evidence that the result was not biased by missing outcome data?**	**Judgment**: We felt that in the context of our review missing outcome data was unlikely to be random: that is missingness was likely to indicate alcohol/drug use. Hence, multiple imputation would not be an appropriate method to correct for missing data, but simple imputation where missing data was considered as relapse was reasonable. Therefore, we coded, ‘Yes/Probably yes’, if simple imputation was used but ‘No/Probably no’ if multiple imputation was used. These decisions are to be made based on the specific review context as there are no general rules. **Implication:** Answering ‘Yes/Probably yes’ meant an outcome could be assessed as ‘Low risk’ of bias. Answering ‘No/Probably no’ meant an outcome could only be assessed as ‘High risk’.
**4.2 Could measurement or ascertainment of the outcome have differed between intervention groups?**	**Judgement:** In some of the interventions a key component was monitoring of alcohol/drug use. Where outcomes were measured by self‐report, we felt it was reasonable to assume that people receiving such interventions would be more able to accurately recall their alcohol/drug use compared to controls. So, if the outcome measure was self‐reported and the intervention involved monitoring of alcohol/drug use, but the control did not, then we coded ‘Yes/Probably yes’. **Implication**: Answering ‘Yes/Probably yes’ rather than ‘no information’ meant an outcome could be assessed as ‘High risk’ of bias rather than ‘Some concerns’.

### Suggestions for Interpreting the Questions in the Review Specific Context

Drawing on our experience, and consistent with the Cochrane editors' recommendations [12], we suggest review teams document review‐specific decisions regarding when it is reasonable to make assumptions when faced with gaps in trial reporting and how to interpret the signaling questions in the context of their review (see Table 1 for an example of selected signaling questions). Providing a template for review teams to record and share review‐specific decisions about how to interpret the questions on the RoB 2 web page would encourage reviewers to be consistent and transparent when interpreting the questions to their review context.

## 5 Conclusions

In this research brief, we reflect on our experience in using RoB 2 in a non‐Cochrane systematic review. Based on our experiences, we make suggestions for review teams and for tool developers to support the application of RoB 2 (see Table 2). Incorrect application of RoB 2, or poor reporting of assessments, call into doubt the assessment process and undermine confidence in the use of evidence to inform policy. Improving the application and reporting of RoB 2 assessments would benefit both evidence syntheses and the decisions that are informed by them. Continuing discussion of RoB 2's application will benefit its refinement and help future users. Reflections from other review teams would be of interest.

**Table 2:** Summary of suggestions when using RoB 2.

Suggestions for review teams
Engage thoughtfully with RoB 2 guidance and other available resources. Recognise that you may face challenges in applying the tool to your review context. Be transparent about the decisions and interpretations made in the process of applying the RoB 2 tool to your specific review, including how you have selected outcome(s) and results for synthesis where these can't be pre‐specified at the protocol stage. Pilot the tool on a sample of results with the full team before formally coding. Hold regular team meetings to discuss emerging challenges. Document and report all decisions and assumptions made when applying RoB 2 to your review.

Suggestions for RoB 2 tool developers
Provide or link to guidance on pragmatic but unbiased approaches for selecting outcomes when pre‐specification at the protocol stage has not occurred or is not feasible. Mention that review teams could sometimes reuse information across assessments. Provide more examples of systematic reviews of complex public health interventions and guidance around when it is reasonable to make assumptions where there are gaps in trial reporting. Provide a template for review teams to record and report review‐specific assumptions and decisions. Consolidate guidance into a single, multi‐layered interactive resource to enable reviewers to link between relevant sections of different resources.

## Author Contributions

**Preethy D'Souza:** conceptualization, investigation, writing – original draft, methodology, validation, writing – review and editing, software, formal analysis, data curation, supervision, resources. **Wendy Macdowall:** conceptualization, investigation, writing – original draft, methodology, validation, writing – review and editing, software, formal analysis, data curation. **Helen E. D. Burchett:** conceptualization, investigation, writing – original draft, methodology, validation, writing – review and editing, software, formal analysis, data curation. **Irene Kwan:** conceptualization, investigation, writing – original draft, methodology, validation, writing – review and editing, software, formal analysis, data curation. **Katy Sutcliffe:** conceptualization, investigation, funding acquisition, writing – original draft, methodology, validation, writing – review and editing, software, formal analysis, project administration, data curation, supervision.

## Conflicts of Interest

The authors declare no conflicts of interest.

## Data Availability

The data that support the findings of this study are available from the corresponding author upon reasonable request.
